# Comment on “The potential pitfalls of synovial sarcoma mimicking intraneural ganglion cyst: A case report and literature review”

**DOI:** 10.1016/j.ijscr.2023.108892

**Published:** 2023-10-01

**Authors:** Kouhei Mitsui, Jiro Ichikawa, Tomonori Kawasaki, Kojiro Onohara

**Affiliations:** a Department of Orthopaedic Surgery, Interdisciplinary Graduate School of Medicine, University of Yamanashi, Yamanashi, Japan; b Department of Radiology, University of Yamanashi, Yamanashi, Japan; c Department of Pathology Saitama Medical University International Medical Center, Saitama, Japan

**Keywords:** Synovial sarcoma, Clinical symptoms, Magnetic resonance imaging, Differential diagnosis, Treatment

We read the article by Dr. Almodumeegh and colleagues titled “The potential pitfalls of synovial sarcoma mimicking intraneural ganglion cysts: A case report and literature review” in the *International Journal of Surgery Case Reports* with great interest [1]. The study emphasizes the clinical pitfalls of synovial sarcoma (SS) with atypical imaging and additional treatment after unplanned excision. We, the sarcoma clinician team, share our opinions and criticisms of this valuable work.

## 1 Details of magnetic resonance imaging

Magnetic resonance imaging (MRI) plays a pivotal role in the differential diagnosis of soft-tissue tumors and is characterized by the presence or absence of contrast effects which is beneficial for differential diagnosis. Additionally, MRI with contrast is beneficial for differentiating cystic lesions from solid tumors [2]. In general, several tumors including benign and malignant tumors occur around the knee. However, in the case of ganglion, enhancement may be seen in the peripheral parts of one of the cystic tumors [2]. In this study, T1-weighted images with contrast, were measured, since another sequence was not available, showed homogenous enhancement. Moreover, the tumor exhibiting the cystic lesion suggested a high signal in T2-weighted images. One of the most important differential diagnoses is benign peripheral nerve sheath tumors (BPNST), including schwannomas and neurofibromas rather than ganglion cysts. High signals in T2-weighted images and enhancement are usually observed in BPNST, which was consistent with this case [3]. However, the target sign, split-fat sign, and fascicular sign, which are characteristic of BPNST, were not observed [3]. Therefore, another differential diagnosis in this case was myxoid tumors, including benign and malignant tumors. In addition, we examined the MRI findings from the perspective of an SS. However, the hallmarks of MRI in SS, triple sign, pseudo-cystic, and tail signs had a positivity of 43 %, 23 %, and 34 %, respectively, suggesting that the positive rate was not very high. Unfortunately, we were unable to confirm these findings in this case [4]. In summary, our team concluded that although this tumor existed as a superficial lesion, a precise differential diagnosis was impossible because both benign and malignant tumors were suspected on MRI. Therefore, we reviewed MRI scans in detail and found that the typical tail sign in the coronal view extended along the fascia in the vertical direction, which is the only finding of suspected sarcoma. The tail sign is often positive in sarcomas, including SS, which may reflect their potential invasiveness. However, although the tail sign in SS is positive in 34 % patients, with no correlation between prognosis and recurrence [4], the tail sign in myxofibrosarcomas is a predictor of recurrence and associated with metastasis at presentation [5]. Therefore, we argue that although MRI and ultrasound are powerful tools for diagnosing soft tissue tumors, ultrasound with Doppler can easily depict the entering and existing nerves, and the intratumoral blood flow to differentiate between cystic and solid tumors [6]. Moreover, if the tumor suspected in this case was only an intraneural ganglion after examination by ultrasound, puncture with caution of nerve damage may have been applicable for the diagnosis and improvement of symptoms. Finally, we reviewed the clinical symptoms, which were not directly connected with MRI because, notably, some cases of SS show neurological symptoms similar to this case [7]. In the present case, the tumor was located above the peroneal nerve, indirectly compressing it via the fascia, resulting in sensory loss and muscle weakness. Although rare, SS originates from the nerve. Therefore, in this situation, with an origin from the nerve, differential diagnosis of peripheral nerve tumors in terms of both clinical symptoms and MRI was extremely difficult [7].

## 2 Pathological findings

In the present case, we recognized the type of operation as an unplanned excision. Usually, the main reason for unplanned excisions is the absence of a pathological examination followed by a pathological diagnosis. We understand that the differential diagnosis from MRI in this case was difficult and limited, therefore, needle biopsy or open biopsy was essential as the next step. SS is defined cytogenetically as the presence of the SS18::SSX 1/2/4 fusion gene [8]. Moreover, immunohistochemistry (IHC) is undoubtedly essential for the diagnosis of SS; however, positive markers, including CD99 and BCL-2 in SS overlap with Ewing sarcoma, making it difficult to confirm the diagnosis [8]. In such cases, the confirmation of fusion genes by Fluorescence in situ hybridization (FISH) or Polymerase chain reaction for the appropriate diagnosis of SS surely helps overcome this limitation of IHC, however, whether FISH can be performed depends on the hospital. Recently, Zaborowski et al. reported the usefulness of IHC using SS18::SSX and SSX antibodies. The positivity rates for SS18::SSX and SSX antibodies in SS were 87.2 and 92.3 %, respectively, with specificities of 100 and 96.1 %, respectively [9]. In addition, the Fédération Nationale des Centres de Lutte Contre le Cancer grade, although not mentioned in this manuscript, is important because it is the main prognostic factor for overall survival [4].

## 3 Treatment

An unplanned excision often occurs in cases with a superficial location and small size [10]. The recurrence rate in unplanned excision is higher than that in planned excision. Moreover, due to the additional wide resection, plastic reconstruction is performed in more cases of unplanned than planned excision [10]. In this case, if we planned an additional wide resection without radiotherapy (RT), the peroneal nerve and fascia would have had to be sacrificed as both had been contaminated by the tumor during the first operation. The range of resection in the deep site, based on the MRI, is at least a part of the muscle, including the tibialis anterior, extensor digitorum longus, fibularis of longus, and gastrocnemius, with a possibility of the fibular collateral ligament and head of the fibula. In addition, plastic reconstructive surgery, including skin flaps or skin grafts may be required in cases where direct wound closure is difficult. Based on these concepts, peroneal nerve palsy was spontaneous in the additional wide resection without RT; however, we understand the authors hypothesized that RT as neoadjuvant therapy would reduce the extent of resection, preserving the peroneal nerve. Although RT is advantageous for the control of local recurrence in SS [11], there have been complications including wound healing followed by infection, osteonecrosis, edema, fibrosis, and nerve damage in the early and late phase [12]. Thus, a high complication rate of RT may have occurred in this case because the tumor was located superficially and was adjacent to the peroneal nerve. Although no nerve damage was observed, a long-term follow-up was required.

## 4 Conclusion

In the diagnosis of some sarcomas, including SS, atypical symptoms and imaging results with pitfalls are occasionally encountered. In such cases, we should return to the principles of diagnosis to complete the process of biopsy, followed by pathological examination.

Although it is important and valuable to avoid missing the tail sign, we must keep the Jaffe's triangle composed of surgeons (clinical features), radiologists (various image findings), and pathologists (pathological findings) in mind.

## Ethical approval

Not applicable.

## Funding

None.

## Author contribution

All authors contributed to the study concept and design, data collection, data analysis and interpretation, and manuscript writing.

## Guarantor

Jiro Ichikawa.

## Patient consent

Not applicable.

## Declaration of competing interest

The authors have no conflicts of interest to disclose.
